# Pectin-Rich Amendment Enhances Soybean Growth Promotion and Nodulation Mediated by *Bacillus Velezensis* Strains

**DOI:** 10.3390/plants8050120

**Published:** 2019-05-09

**Authors:** Mohammad K. Hassan, John A. McInroy, Jarrod Jones, Deepak Shantharaj, Mark R. Liles, Joseph W. Kloepper

**Affiliations:** 1 Department of Entomology and Plant Pathology, Auburn University, CASIC Building, Auburn, AL 36849, USA; mkh0025@auburn.edu (M.K.H.); mcinrja@auburn.edu (J.A.M.); 2 Gulf Coast Research and Extension Center (GCREC), Fairhope, AL 36532, USA; jones39@auburn.edu; 3 Department of Biological Sciences, Auburn University, CASIC Building, Auburn, AL 36849, USA; dzs0081@auburn.edu (D.S.); lilesma@auburn.edu (M.R.L.)

**Keywords:** PGPR, *Bacillus velezensis* (Bv) strains, *Bradyrhizobium japonicum* inoculant, in vitro assay, pectin and orange peel amendments, soybean, field soil, greenhouse tests, field test

## Abstract

Plant growth-promoting rhizobacteria (PGPR) are increasingly used in crops worldwide. While selected PGPR strains can reproducibly promote plant growth under controlled greenhouse conditions, their efficacy in the field is often more variable. Our overall aim was to determine if pectin or orange peel (OP) amendments to *Bacillus velezensis* (Bv) PGPR strains could increase soybean growth and nodulation by *Bradyrhizobium japonicum* in greenhouse and field experiments to reduce variability. The treatments included untreated soybean seeds planted in field soil that contained Bv PGPR strains and non-inoculated controls with and without 0.1% (*w/v*) pectin or (1 or 10 mg/200 μL) orange peel (OP) amendment. In greenhouse and field tests, 35 and 55 days after planting (DAP), the plants were removed from pots, washed, and analyzed for treatment effects. In greenhouse trials, the rhizobial inoculant was not added with Bv strains and pectin or OP amendment, but in the field trial, a commercial *B. japonicum* inoculant was used with Bv strains and pectin amendment. In the greenhouse tests, soybean seeds inoculated with Bv AP193 and pectin had significantly increased soybean shoot length, dry weight, and nodulation by indigenous *Bradyrhizobium* compared to AP193 without pectin. In the field trial, pectin with Bv AP193 significantly increased the shoot length, dry weight, and nodulation of a commercial *Bradyrhizobium japonicum* compared to Bv AP193 without pectin. In greenhouse tests, OP amendment with AP193 at 10 mg significantly increased the dry weight of shoots and roots compared to AP193 without OP amendment. The results demonstrate that pectin-rich amendments can enhance Bv-mediated soybean growth promotion and nodulation by indigenous and inoculated *B. japonicum*.

## 1. Introduction

Plant growth-promoting rhizobacteria (PGPR) colonize the plant rhizosphere and stimulate plant growth through diverse mechanisms such as nitrogen fixation [1], phosphate solubilization [2], siderophore production [3], phytohormone production [4], and the secretion of volatile organic compounds (VOCs) [5]. *B. velezensis* (Bv) (previously known as *Bacillus amyloliquefaciens* subsp. *plantarum*) is a gram-positive, rod-shaped PGPR species that includes strains reported to enhance the plant growth of several plants, including maize [6], soybean [7], oilseed rape (*Brassica napus*) [8], and *Arabidopsis thaliana* [9]. 

In addition to promoting plant growth, many Bv strains inhibit plant pathogens through the secretion of bioactive secondary metabolites and volatile organic compounds (VOCs). Palazzini et al. [10] reported that iturin and fengycin secreted from Bv RC 2018 suppressed *Fusarium* head blight caused by *Fusarium graminearum*. Three volatile organic compounds (pyrazine, benzothiazole, and phenol-2,4-bis) of Bv strain ZSY-1 exhibited antifungal activity against *Alternaria solani* and *Botrytis cinerea* [11]. Our previous comparative genomic study of *B. amyloliquefaciens* and Bv strains [12] predicted 73 genes that were exclusively identified among Bv PGPR strains, including genes involved in carbon source utilization and secondary metabolite production. Interestingly, this previous study predicted that all of the Bv PGPR strains for which genome sequences were available (*n* = 28) could degrade pectin and also utilize it as a sole carbon source. Hence, in the current study, we screened a collection of 59 Bv PGPR strains for the capacity to use purified pectin as a sole carbon source to determine if this is a conserved trait among plant growth-promoting Bv strains. 

Pectin has multiple functions in plant growth, morphology, plant development, cell expansion, seed hydration, and plant defense [13,14]. Pectin is present in the peel of several fruits, including apple [15], passion fruit [16], and orange [17,18]. The separation of root border cells from the root cap is induced by pectin methylesterase activity [19], resulting in the release of soluble, de-esterified pectin that can have multiple impacts on bacterial-mediated plant growth-promotion and plant health [20]. The soluble pectin produced by root border cells could be used as a nutrient source by rhizobacteria [19,21]. While pectin is found in various plant tissues such as the root tip, leaves, and fruits, the highest percentage of pectin occurs in fruit. For example, orange peel contains 30% pectin [22], making it the preferred source of pectin [23]. There are currently only three commercially available sources of pectin extracts in the United States (U.S.): sugar beet [24], apple pomace [25], and citrus peel [26]. A recent study conducted by Wu et al. [27] demonstrated that exogenous pectin or other carbohydrate amendments induced *B. amyloliquefaciens* SQY 162 to increase biofilm formation and secretion of the secondary metabolite surfactin, resulting in enhanced biocontrol activity against *Ralstonia solanacearum* in tobacco. However, this study did not investigate the potential synergy between pectin and PGPR-mediated plant growth-promotion. Hence, the overall objective of this study was to test the hypothesis that pectin-rich amendments enhance the plant growth-promoting effects of Bv PGPR strains on soybean. 

## 2. Materials and Methods

### 2.1. Bacterial Strains and Culture Conditions

Fifty-nine Bv strains were previously isolated and identified as being affiliated with Bv based on 16S rRNA and *gyrB* gene sequences, and each strain had been previously shown to have PGPR activity [12,28,29,30]. Bv strains were transferred from cryostocks at −80 °C onto tryptic soy agar (TSA) and were incubated at 28 °C for 24 h. A single colony of each strain was streaked onto spore preparation medium [31] and incubated at 28 °C for seven days. Sterilized distilled water (15 mL) was added to each plate, and the cellular mass was transferred to a 50-mL centrifuge tube. Bv suspensions were heat-treated at 80 °C for 20 min, serially diluted, and adjusted to 1.0 × 10^6^ spore colony-forming units (CFU)/mL. 

### 2.2. Pectate Lyase Activity Test

Bacteria were cultured from cryostocks at −80 °C into tryptic soy broth (TSB) at 28 °C overnight using 220 rpm for 5-mL culture. A one-ml aliquot was pipetted into a 1.5-mL microcentrifuge tube, and tubes were centrifuged for 5 min at 10,000 × *g* speed. The supernatant was discarded, and the process was repeated three times using sterile water. To the final bacterial pellet, 1.0 mL of sterile water was added to each microcentrifuge tube and vortexed thoroughly to produce a uniform bacterial suspension. A 1.0-mL aliquot of each strain was transferred to a cuvette to measure turbidity, adding sterilized water until the optical density at 600 nm (OD_600_) was approximately 0.5. Twenty μL of this standardized bacterial suspension was transferred in triplicate onto pectate-agar (Pa) medium [32] to determine the pectate lyase activity. Tris-HCl buffer was adjusted (0.1M, pH 8.0) for the Pa medium separately and sterilized using a 0.45-μm Nalgene syringe filter (Thermo Scientific, Waltham, MA, USA). The Pa medium plates were incubated at 28 °C for 24 to 48 h, and then 1% cetyltrimethyl ammonium bromide (CTAB) was poured over the surface of each plate at room temperature. The resulting pectin clear zones were measured in millimeters (mm), and pectate lyase activity (PLA) was rated on a scale of low (OD_600_ 0.1–0.2) (+), medium (OD_600_ 0.2–0.4) (++), and high (OD_600_ 0.4–0.6) (+++). 

### 2.3. Growth of Bv PGPR Strains Using Pectin as a Sole Carbon Source

Each Bv strain was assessed for its ability to utilize pectin as a sole carbon source using a Tris-Spizizen Salts (TSS) [33] minimal medium supplemented with 0.1% pectin powder (EC No. 232-553-0, Tokyo Chemical Industry Co., Toshima, Kita-Ku, Tokyo, Japan). The TSS minimal medium was filter sterilized using a 0.45-µm polyethersulfone (PES) vacuum filter unit (VWR, Radnor, PA, USA). Each of the bacterial cultures was grown overnight in TSB medium, and the cell pellets were washed three times in sterilized water, normalized to OD_600_ = 0.5, and then 100 µL of a 1:100 dilution was used to inoculate 1.9 mL TSS+0.1% pectin cultures to adjust the OD_600_ = 0.030, in triplicate. Bacterial cells were grown at 28 °C with 200 rpm continuous shaking for 72 h in a shaking incubator, and readings at OD_600_ were recorded.

### 2.4. Greenhouse Trials of Pectin and PGPR Amendments on Soybean to Assess Root Colonization, Growth Promotion, and Nodulation

#### 2.4.1. Preparation of Pectin Powder and Liquid Suspensions 

Pectin powder (from the citrus peel source, described above) was mixed thoroughly with field soil using a soil mixer at a rate of 1.0 g per 1000 g of field soil. In addition, pectin powder (0.1 g) was suspended with sterilized water at a rate of 1.0 g per 1000 mL water for application as an aqueous pectin suspension.

#### 2.4.2. Field Soil Preparation

Sandy loam field soil collected from the E.V. Smith Research Center (Shorter, AL), and sieved to remove root debris, was used for the greenhouse experiment. Soil (450 g) was placed in each cone-tainer tube (lightweight large Deepots D40L, Stuewe & Sons, Danville, IL, USA) that contained three cotton balls in the bottom to retain soil. 

#### 2.4.3. Soybean Seed Inoculation

Soybean seed variety (‘Progeny P5333 RY’) not treated with chemicals was used for all of the greenhouse experiments. One ml of Bv PGPR strains (1.0 × 10^6^ spore CFUs/mL) was pipetted over each seed. Two seeds were placed in each cone-tainer to ensure germination, and one seedling was removed one week after planting. Then, 5.0 g of soil was placed over each seed. Each cone-tainer rack was covered by a plastic sheet for 48 h to prevent soil desiccation. Afterward, racks were transferred to the greenhouse and tubes were watered twice daily. 

#### 2.4.4. Soybean Plant Growth Measurement

At 35 days after planting (DAP), all the plants were harvested for plant morphometric measurements. Shoot length was measured from the growing apical region to the basal region connected to the root. Root length was measured from the root tip to the basal region connected to the root. For dry weight measurements, shoots and roots were oven dried at 70 °C for 48 h. 

#### 2.4.5. Selection of Bv Rifampicin-Resistant Mutants and Evaluation of Bv PGPR Strains Root Colonizing Capacity 

Three strains (AP136, AP143, and AP193) were streaked onto TSA plates for 24 h to ensure the purity of the bacterial colony. From each strain, one colony was transferred into 30-mL TSB in a sterile 50-mL centrifuge tube, and placed in a shaking incubator (220 rpm) at 28 °C. Rifampicin (Sigma, St. Louis, MO, USA) antibiotic was used for the selection of Bv mutants. To prepare the stock solution of 50 mg/mL of rifampicin (Sigma-Aldrich, Product code 101594249, St. Louis, MO, USA), 500 mg of rifampicin (rif) was added to 10 mL of dimethyl sulfoxide (DMSO). The stock solution was sterilized using a 0.45-μm Nalgene syringe filter (Thermo Scientific, Waltham, MA, USA). After 24 h, 50 μg/mL of rifampicin working concentration was added to 50 mL of TSB bacterial culture media. The rif–TSB culture tube was wrapped with aluminum foil to prevent the degradation of rifampicin by light and placed in a shaking incubator at 28 °C. After 48 h, one loop from each rif–TSB culture tube was streaked onto a TSA+rif plate of each strain and placed into the incubator at 28 °C. Single colonies that grew on TSA+rif plates were removed, labeled as AP136-rif, AP143-rif, and AP193-rif, and placed into the −80 °C freezer. At 35 DAP, the populations of Bv PGPR populations in the soybean rhizosphere were evaluated. The adherent soil was removed gently from the roots of each plant and placed in 15-mL screw-cap tubes. Nine ml of sterile water was added to each tube, and the tubes were vortexed thoroughly. Then, serial dilutions were made from 1:10 to 1:1000 in sterile Milli-Q water in microcentrifuge tubes, and 100 µL was plated on TSA+rif plates for each dilution and incubated at 28 °C for 24 to 48 h. Colonies that grew on the TSA+rif plates that had the same colony morphology as the inoculated strain were counted and expressed in log CFU/mL.

#### 2.4.6. Evaluation of Soybean Nodulation

At 35 DAP, soybean nodules per plant were removed from the roots, counted, and oven dried in a mechanical convection oven at 70 °C for 48 h. Then, the nodule dry weight was recorded.

### 2.5. Field Trial of Pectin and PGPR Amendments on Soybean Growth Promotion and Nodulation

#### 2.5.1. Soil Type and Application of *Bradyrhizobium Japonicum* Inoculant 

The field trial was conducted in the Alabama Agricultural Experiment Station’s (AAES) Gulf Coast Research and Extension Center (GCREC) located in Fairhope, Alabama (AL). GCREC Soil was a Malbis fine sandy loam type. A commercial *B. japonicum* inoculant powder (HiStick N/T, BASF, North Carolina, NC, USA) was directly applied to the seed at planting via a hopper-box treatment into the furrow. According to the label, the population of *B. japonicum* inoculant was (2.0 × 10^9^ cells/g). 

#### 2.5.2. Soybean Seed Inoculation

Soybean seed variety “Asgrow 75×6” was used for the field trial. The soybean seeds were planted directly from a hopper box into each plot. Each plot had four rows. A Bv spore suspension at 1.0 × 10^6^ spore CFU/mL was applied in-furrow at the rate of 37.85 liters per hectare, and pectin liquid suspension (0.1%) was sprayed over seeds at the time of planting. 

#### 2.5.3. Soybean Plant Growth Measurement

Soybean shoot length was assessed at 35 and 55 days after planting (DAP). Soybean dry shoot weight was measured after drying in an oven at 70 °C for 48 h at 35 and 55 DAP. Soybean yield was assessed by harvesting at 140 DAP, and soybean seed weights were recorded from the two center rows of each four-row plot. 

#### 2.5.4. Evaluation of Soybean Nodulation

Treatment effects on soybean nodulation were determined by counting the number of nodules per plant and by measuring the total dry weight of nodules per plant at 35 DAP. To assess nodule dry weight, nodules were removed from roots and dried in a mechanical convection oven at 70 °C for 48 h. 

### 2.6. Greenhouse Trials of Orange Peel Liquid Suspension and PGPR Amendments on Soybean Growth Promotion and Nodulation in Field Soil

#### 2.6.1. Growth of Bv PGPR Strains Using Orange Peel as a Sole Carbon Source

The same methods were followed for the in vitro Bv strain growth experiments using orange peel powder as a sole carbon source. Organic orange peel powder was collected from Citrus Extracts (Fort Pierce, FL 34982, USA). 

#### 2.6.2. Preparation of Orange Peel Liquid Suspensions

Orange peel powder (500 mg) was used for the greenhouse tests (Citrus Extracts, Fort Pierce, FL 34982, USA), which was added into 10 mL of sterilized water until thoroughly dispersed and applied (1 or 10 mg/200 μL) onto the soybean seed surface after inoculation with the Bv strains as described above. 

#### 2.6.3. Field Soil Preparation

A sandy loam field soil was collected and prepared for orange peel liquid suspensions test by the same methods described above. The same amount of soil was placed in each cone-tainer tube that contained three cotton balls in the bottom to retain soil. 

#### 2.6.4. Soybean Seed Inoculation 

A soybean seed variety (‘Progeny P5333 RY’) without chemical seed treatments was used for the greenhouse experiment. Two seeds were placed in each cone-tainer to ensure germination, and one seedling was removed one week after planting. Orange peel suspensions (1 or 10 mg/200 µL) and 50 µL of Bv strains (1.0 × 10^6^ spore CFUs) were pipetted separately over each seed. Then, 5.0 g of soil was placed over each seed. Each cone-tainer rack was covered by a plastic sheet to prevent soil desiccation for 48 h. Afterward, cone-tainer racks were transferred to the greenhouse and were watered twice daily. 

#### 2.6.5. Soybean Plant Growth Measurement and Nodule Evaluation

The soybean plant growth parameters and the numbers of nodules per plant were assessed by the same methods that were followed for the pectin experiments in the greenhouse tests. 

### 2.7. Statistical Analyses and Experimental Design

In the greenhouse tests, cone-tainers were arranged in a randomized complete block design (RCBD) with eight treatments and 12 replications, with each replication being a single plant in a single cone-tainer. The data of mean shoot height, mean root length, mean dry shoot weight, mean dry root weight, a mean number of root nodules per plant, and rhizobacterial CFUs were analyzed with SAS 9.4 software (SAS Institute, Cary, NC, USA) using the PROC GLIMMIX. Treatment means were compared using LSMEANS at the *p* < 0.05 level of significance. 

In the field test, the experiment design was a 6 × 6 Latin square design with 36 total plots. Each plot consisted of four 9.1-m long rows. The planting rate was 120,000 seeds per acre or eight seeds per 0.3 m of row. The data of plant mean shoot height, mean dry shoot weight, mean nodule numbers per root, and mean nodule dry weight were analyzed with SAS 9.4 software (SAS Institute, Cary, NC, USA) using Duncan’s multiple range test at the 5% level of significance. 

## 3. Results

Pectinase clear zones appeared around the colonies of all the tested Bv strains after 30 min (Table 1). Very strong pectate lyase activities were observed for strains AP52, AP80, AP81, AP87, AP112, AP143, AP183, AP188, AP190, AP191, AP192, AP199, AP200, AP203, AP207, AP208, AP212, AP296, AP298, and AP299, while all other Bv strains exhibited pectate lyase activity albeit to a lesser degree (Table 1).

**Figure 1:**
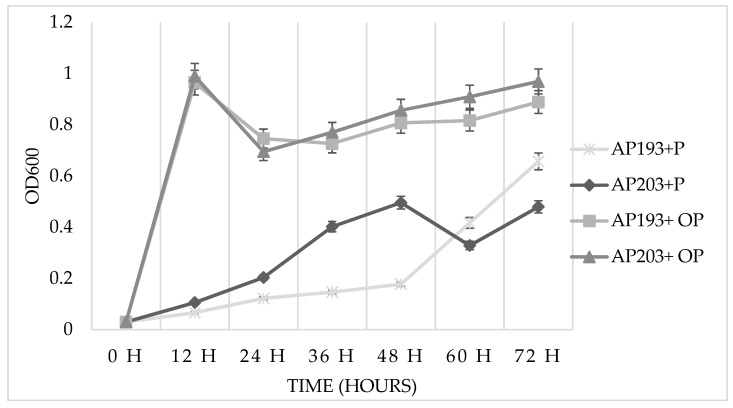
In vitro growth assay of *B. velezensis* (Bv) plant growth-promoting rhizobacteria (PGPR) strains in Tris-Spizizen Salts (TSS) minimal medium including 0.1% (*w/v*) pectin powder (P) or 0.5% (*w/v*) orange peel (OP) amendments.

All of the 59 Bv PGPR strains grew well in TSS minimal medium containing purified pectin as a sole carbon source (Table 1 and Figure 1). The highest OD_600_ values were observed for Bv PGPR strains AP67, AP71, AP75, AP77, AP78, AP85, AP87, AP108, AP112, AP135, AP135, AP143, AP183, AP184, AP188, AP191, AP192, AP193, and AP203. Based on their observed ability to grow in vitro using pectin as a sole carbon source, the four best-growing Bv PGPR strains AP136, AP143, AP193, and AP203 were selected for greenhouse and field trials. 

**Figure 2:**
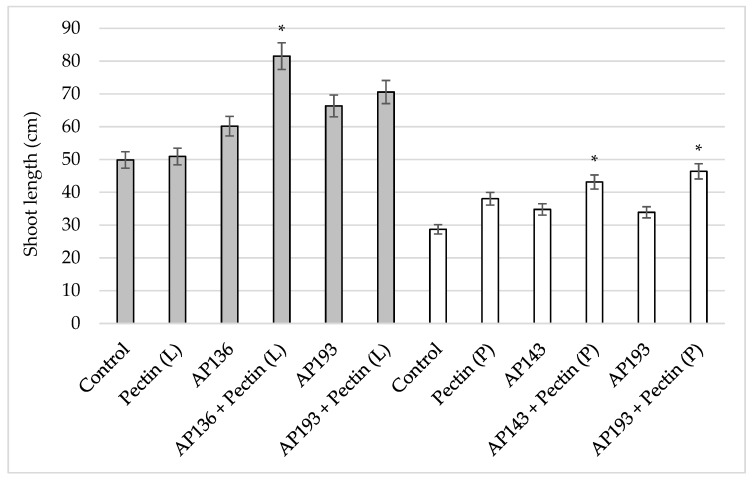
Effect of pectin powder or liquid suspension amendments on soybean shoot length by Bv PGPR strains at 35 DAP^#^ in the greenhouse trials. The gray bar indicates amendment with 0.1% (*w/v*) pectin liquid suspension (L), while the white bar indicates amendment with the comparable amount of pectin powder (P) (* indicates significance at the 5% level relative to the Bv PGPR strains alone) (DAP^#^—days after planting).

Soybean shoot length was significantly enhanced by the combination of purified pectin powder amendment and inoculation with Bv AP193 relative to the shoot length observed when Bv AP193 was applied in the absence of pectin (Figure 2); however, the same enhanced shoot length was not observed with the combination of pectin liquid and Bv AP193 (Figure 2). The best result was observed when pectin was applied as a liquid suspension, with the combination of Bv AP136 and pectin showing a significant increase in shoot length compared to Bv AP136 alone (Figure 2). Mean root lengths were not significantly different when comparing Bv strains AP193 and AP143 with and without pectin powder amendment (Table 2). Pectin powder amendment with Bv PGPR strain AP193 significantly increased dry shoot weight compared to Bv PGPR strain AP193 without pectin powder amendment (Table 2). 

**Figure 3:**
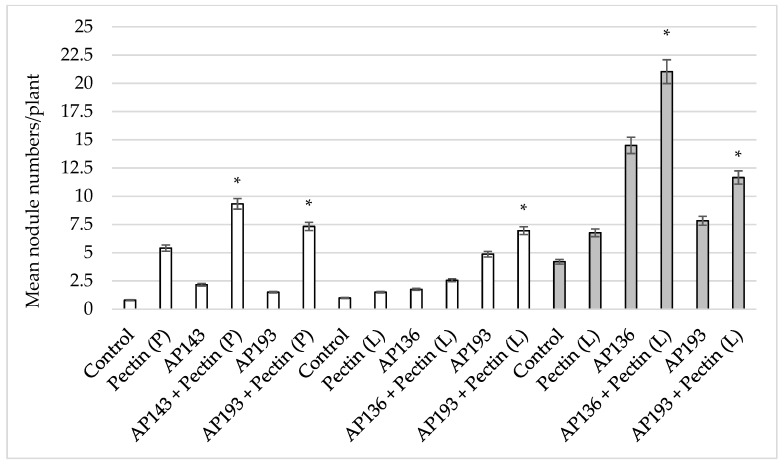
Effect of pectin powder or liquid suspension amendments on soybean nodulation by Bv PGPR strains at 35 DAP^#^ in the greenhouse and field trials. The gray-colored bar indicates the results of the field trial with a 0.1% (*w/v*) pectin liquid suspension, and the white-colored bar indicates greenhouse trials in which pectin was applied as a powder (P) or as a liquid suspension (L) amendment (* indicates significance at the 5% level relative to the Bv PGPR strains alone) (DAP^#^—days after planting).

Interestingly, a significant increase in soybean nodulation was observed when seeds were inoculated with both purified pectin and Bv spores (Figure 3). When pectin and Bv strains were applied together, there was a significant increase in nodules, of 331% and 388%, when pectin was applied as a powder together with Bv AP143 or Bv AP193, respectively (Figure 3). Application of purified pectin as a powder or liquid suspension together with Bv AP193 similarly induced a significant increase in the number of nodules per plant (Figure 3). In each of these experiments, the nodules were formed either by infection with indigenous rhizobia in the field soils or by commercial *B. japonicum* inoculant. While significant effects on nodulation due to *B. japonicum* were observed, the populations of the inoculated Bv PGPR strains did not significantly change as a result of pectin amendment (Table 2).

**Figure 4:**
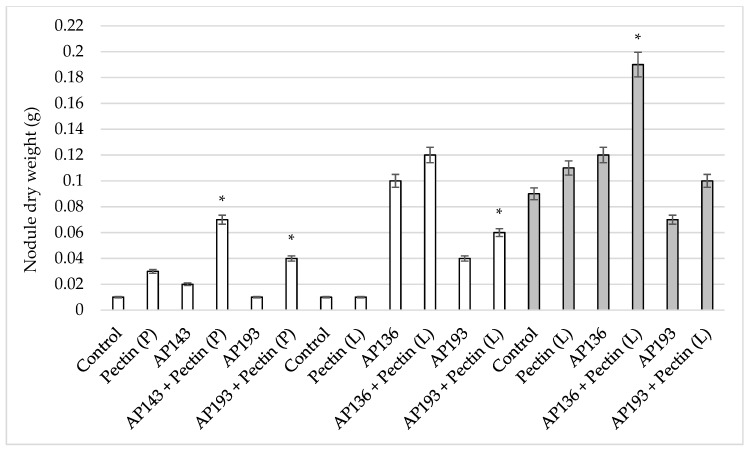
Effect of pectin powder or liquid suspension amendments on soybean dry nodule weight by Bv PGPR strains at 35 DAP^#^ in the greenhouse and field trials. The gray-colored bar indicates the results of the field trial with a 0.1% (*w/v*) pectin liquid suspension, and the white-colored bar indicates greenhouse trials in which pectin was applied as a powder (P) or as a liquid suspension (L) amendment (* indicates significance at the 5% level relative to the Bv PGPR strains alone) (DAP^#^—days after planting).

In the field trial, the treatment of soybean seeds with Bv AP193 and a pectin liquid suspension resulted in a significant increase in shoot length and dry shoot weight at 55 DAP (Table 3). The inoculation of seeds with Bv AP193 resulted in a mean shoot length of 57.2 cm and a mean dry shoot weight of 14.4 g, whereas an inoculation with both pectin and Bv AP193 resulted in an average shoot length of 78.7 cm (37.6% increase) and an average dry shoot weight of 16.93 g (17.6% increase). The mean number of nodules on plants treated with Bv AP136 or AP193 and a pectin amendment were significantly more than on plants seeds treated with these Bv strains alone (Figure 3). The nodule dry weight of plants treated with Bv AP136 and pectin was significantly greater than the Bv AP136 control alone (Figure 4). The effect of pectin amendment on soybean yield was also assessed in the field trial, with no significant differences observed among the different treatment groups for plot or test weights (Table 4). The combination of Bv strain and pectin amendment did not increase soybean yield over the Bv strains alone, pectin, or water control, nor did soybean yield change in response to pectin amendment compared to the water control. 

The lack of consistent plant growth-promoting effects using purified pectin as an amendment in greenhouse and field trials led to the evaluation of orange peel powder as a pectin-rich organic amendment. To first evaluate Bv strains’ in vitro growth using orange peel powder as a growth substrate, Bv strains were inoculated into the TSS minimal medium. A rapid growth of each Bv strain was observed compared to growth using purified pectin as a sole carbon source (Figure 1). 

**Figure 5:**
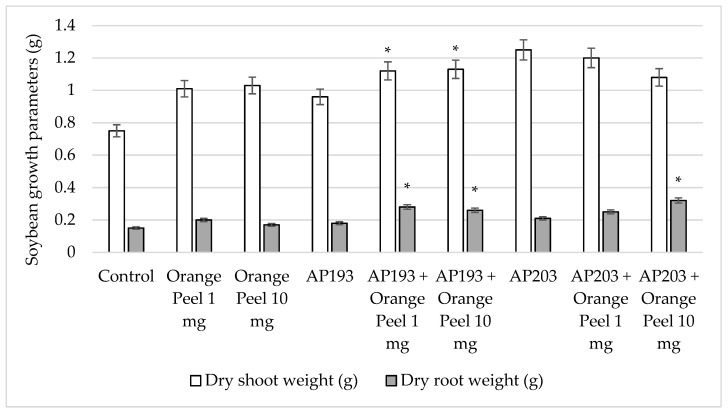
Effect of orange peel (OP) amendments on soybean dry shoot (white bar graph) and root weights (gray bar graph) by Bv PGPR strains at 35 DAP in the greenhouse tests (* indicates significance at the 5% level relative to the Bv PGPR strains alone) (DAP^#^—days after planting).

Given the much greater growth rate observed for Bv strains in vitro on a pectin powder growth substrate, it was of interest to evaluate the combination of Bv spores and orange peel powder as a seed amendment. A greenhouse trial using field soil was conducted as before, but in this case, the amendment consisted of orange peel powder as a liquid suspension with two different doses (1 or 10 mg) applied with or without Bv inoculum (Table 5). At 35 DAP, the mean dry shoot weights of Bv AP193 with orange peel amendment at 1 mg or 10 mg were significantly increased compared to that of Bv AP193 alone (Figure 5). Similarly, the dry root weights were significantly greater when seeds were amended with Bv AP193 with either rate of orange peel amendment compared to Bv AP193 alone (Figure 5). The dry root weights and the numbers of nodules per plant also increased significantly when Bv AP203 was applied together with orange peel (10 mg) compared to plants that received Bv AP203 without orange peel amendment (Figure 5). 

## 4. Discussion

The results of the in vitro growth assays indicated that Bv strains could degrade and utilize exogenous pectin or pectin-rich citrus peel as a sole carbon and energy source. While a slow rate of growth was observed when purified pectin was added to a minimal medium, the *B. velezensis* growth observed in the presence of orange peel powder was significantly greater, suggesting that additional nutritional requirements for Bv growth were supplied from the orange peel powder. A previous study [27] assessed the effects of adding different carbohydrates to the complex medium Lysogeny broth (LB) on the growth of *B. amyloliquefaciens* SQY 162 (a strain that may now be affiliated with *B. velezensis*), and did not observe any significant differences in growth for any added carbohydrate; however, due to the use of the nutrient-rich LB medium in these experiments, this precluded any assessment of *Bacillus* growth due to the use of any of these carbohydrates as a sole carbon and energy source. Hence, our study is therefore the first demonstration of the growth of *B. velezensis* strains using pectin or a pectin-rich organic source such as orange peel powder as a substrate. Given the ubiquity of *B. velezensis* strain growth using a pectin substrate, this suggests that pectin utilization is an important function among these rhizobacteria that may be important in their root colonization and plant growth-promoting activities. The overall results of this study support the hypothesis that pectin amendment can enhance plant growth promotion mediated by selected Bv strains. Plant responses to pectin amendment depended on the specific PGPR strain, and how pectin was inoculated onto a soybean seed. We observed differences in our results depending on whether the purified pectin was applied as a powder or a liquid suspension, and we suspect that Bv strains may have utilized the pectin applied as a liquid suspension more efficiently because of increased bioavailability. There were also differences observed concerning Bv strain performance. For example, Bv strain AP193 was one of the best-performing strains in this study, causing significant increases in the shoot length of soybean plants observed after treatment together with pectin and in a pectin-rich orange peel powder. While Bv strain AP193 was one of the strains observed to grow very well in a minimal medium containing either pectin or orange peel powder, other Bv strains showed comparable in vitro growth and yet did not perform as well in plant trials, suggesting that other bacterial functions such as secondary metabolite biosynthesis might explain these strain differences. A previous study [27] demonstrated that pectin increased biofilm formation, chemotactic activity, and extracellular polymeric substance (EPS) production by *B. amyloliquefaciens* SQY 162, resulting in the enhanced root colonization of tobacco. In our study, there were no significant increases in Bv populations in plant rhizospheres despite significant effects observed on plant physiology. This suggests that pectin-rich amendments are used by Bv strains in the rhizosphere for the production of bacterial products such as EPS or secondary metabolites that can have effects on plant growth promotion, rather than simply being used to increase bacterial populations as was observed in vitro. 

Soybean growth parameters varied with the pectin source, the doses of application, and individual Bv strains’ performance in field soils. For example, while Bv strain AP193 combined with either 1 or 10 mg of orange peel amendment significantly increased the dry shoot and root weights, the synergy between Bv strain AP203 and orange peel powder only occurred at the 10-mg dose. These results suggested that different Bv strains degrade and utilize pectin differently based on pectin sources. In addition, the composition of the pectin-rich amendment, such as phenolic compounds from orange peel [40,41], may also influence the PGPR-mediated induction of soybean plant growth. Citrus fruit peel contains a large amount of pectin [42], and Treuer et al. [43] demonstrated that the application of pectin-rich orange peel to soils provided a long-term benefit for the soil and increased forest vegetation in Costa Rica. Given that different citrus peel sources have different compositions of phenolic, carbohydrate, and other chemical moieties, this could affect the PGPR strain response to these organic amendments; therefore, in a preliminary study, we evaluated different citrus peel powders for their synergy in promoting soybean growth and observed comparable results between orange, grapefruit, lemon, and tangerine peel powders (data not shown). Due to the relatively low cost of orange peel powder compared to other pectin-rich amendments, we selected orange peel powder as the most practical and sustainable amendment for these studies. Interestingly, we consistently observed enhanced nodulation in soybean amended with Bv strains and either purified pectin or orange peel powder. Soybean root nodulation was significantly greater when both pectin and Bv spores were applied, compared to spores alone, and these results were observed in both greenhouse and field trials and with pectin applied as a powder or in liquid suspension. These results strongly indicate that pectin mixed with Bv strains can induce soybean nodulation by indigenous and by inoculated rhizobia. Masciarelli et al. [44] reported that a mixed inoculation of *B. japonicum* with *B. amyloliquefaciens* strain LL2012 enhanced soybean nodulation. Another study indicated that *Bacillus cereus* UW85 increased soybean nodulation in a growth chamber and under field conditions without the inoculation of *Bradyrhizobium* spp. [45]. These reports collectively support the conclusion that there are synergistic interactions between *Bradyrhizobium* spp. and some *Bacillus* spp. that either directly or indirectly (e.g., by Bv interactions with plant root cells) result in enhanced soybean root nodulation. The results of our study support these previous conclusions, and also indicate the role of complex carbohydrates such as pectin in enhancing these rhizobacteria–plant interactions. Further research should explore the molecular interactions between Bv PGPR strains and rhizobia in promoting legume infection and nodulation processes.

In conclusion, the results of this study indicate the importance of pectin as a complex carbohydrate that can be utilized by Bv PGPR strains, and that the exogenous application of pectin-rich amendments can enhance soybean growth and *Bradyrhizobium* nodulation. Future studies are required to extend our understanding of the use of pectin-rich amendments in synergy with select Bv PGPR strains to enhance plant growth, legume nodulation, and/or disease control under field conditions. 

## Figures and Tables

**Table 1:** In vitro degradation and utilization activities of pectin as a sole carbon source for growth for each Bv strain.

Bv Strain	*Pectate Lyase Activity	OD_600_	Reference
AP52	+++	0.36	Kumar et al., 2011 [34]
AP67	++	0.51	This study
AP71	++	0.51	Hossain et al., 2015 [12]
AP75	++	0.49	This study
AP76	++	0.39	This study
AP77	++	0.55	This study
AP78	++	0.49	This study
AP79	++	0.4	Hossain et al., 2015 [12]
AP80	+++	0.32	This study
AP81	+++	0.35	This study
AP85	++	0.57	This study
AP86	++	0.4	This study
AP87	+++	0.45	This study
AP108	++	0.66	This study
AP112	+++	0.52	This study
AP135	++	0.52	This study
AP136	++	0.44	Liu et al., 2016 [29]
AP143	+++	0.49	Coy et al., 2017 [28]
AP150	++	0.35	This study
AP183	+++	0.54	Nasrin et al., 2015 [35]
AP184	++	0.6	This study
AP188	+++	0.72	Zebelo et al., 2016 [36]
AP189	++	0.37	This study
AP190	+++	0.27	This study
AP191	+++	0.67	This study
AP192	+++	0.66	This study
AP193	++	0.68	Ran, 2013 [37]
AP194	++	0.33	Liu et al., 2016 [29]
AP195	++	0.36	Liu et al., 2016 [29]
AP196	++	0.34	This study
AP197	++	0.38	Liu et al., 2016 [29]
AP198	++	0.35	This study
AP199	+++	0.29	Liu et al., 2016 [29]
AP200	+++	0.24	Liu et al., 2016 [29]
AP201	++	0.33	Liu et al., 2016 [29]
AP203	+++	0.46	Liu et al., 2016 [29]
AP205	++	0.34	This study
AP207	+++	0.24	This study
AP208	+++	0.38	Liu et al., 2016 [29]
AP210	++	0.15	Liu et al., 2016 [29]
AP211	++	0.2	This study
AP212	+++	0.22	Liu et al., 2016 [29]
AP213	++	0.29	Liu et al., 2016 [29]
AP214	++	0.2	Liu et al., 2016 [29]
AP215	++	0.09	This study
AP216	++	0.38	This study
AP218	+	0.1	Coy et al., 2017 [28]
AP219	++	0.21	Kumar et al., 2011 [34]
AP241	++	0.1	This study
AP260	++	0.17	This study
AP295	++	0.18	Liu et al., 2016 [29]
AP296	+++	0.11	This study
AP297	++	0.22	Liu et al., 2018 [38]
AP298	+++	0.22	Liu et al., 2018 [38]
AP299	+++	0.19	This study
AP300	++	0.05	This study
AP301	++	0.09	Yellareddygari et al., 2014 [39]
AP304	++	0.2	Kumar et al., 2011 [34]
AP305	++	0.11	Liu et al., 2016 [29]

* Pectate lyase activity were rated on a scale of low (OD_600_ 0.1–0.2) (+), medium (OD_600_ 0.2–0.4) (++), and high (OD_600_ 0.4–0.6) (+++).

**Table 2:** Effect of pectin powder or liquid amendments on soybean growth, nodulation by indigenous soil rhizobia, and root colonization by Bv PGPR strains in greenhouse tests at 35 days after planting (DAP^#^). The mean values in the column followed by the same letter are not significantly different at *p* ≤ 0.05 using Tukey’s multiple comparison tests.

Treatment	Dry Shoot Weight (g)	Root Length (cm)	Dry Root Weight (g)	Root Colonization^#^ (log CFU/g)
Control	0.4bc	24.2a	0.07cd	1.1c
Pectin Powder (0.1%)	0.3d	18.6b	0.07cd	2.4c
AP143	0.4b	22.9a	0.16a	3.7b
AP143+ Pectin Powder (0.1%)	0.6ab	22.8ab	0.15ab	4.1ab
AP193	0.4bc	25.3a	0.10bc	4.5a
AP193+ Pectin Powder (0.1%)	0.6a	26.4a	0.15ab	4.9a
Control	0.9a	20.7b	0.23b	1.2c
Pectin Liquid (0.1%)	0.1a	23.1ab	0.22b	6.5b
AP136	1.3a	28.3ab	0.21a	7.7a
AP136+ Pectin Liquid (0.1%)	1.1a	30.8a	0.27a	7.8a
AP193	1.7a	28.8ab	0.34a	7.4a
AP193+ Pectin Liquid (0.1%)	1.8a	32.1a	0.40a	7.4a

**Table 3:** Effect of pectin amendment when applied as a liquid suspension on soybean plant growth and nodulation by *B. velezensis* (Bv) PGPR strains AP136, AP193, and commercial *B. japonicum* inoculant in the field trial. Note that for all the treatment groups, pectin was applied as a liquid suspension at 1% (*w/v*). The mean values in the columns followed by the same letter are not significantly different at *p* ≤ 0.05 using Duncan’s multiple range tests (DAP^#^—days after planting).

Treatment	DAP^#^	Shoot Length (cm)	Dry Shoot Weight (g)	Root Length (cm)	Dry Root Weight (g)
Control		37.4b	2.6ab	14.5c	0.7ab
Pectin		41.9b	3.6ab	16.6bc	0.7ab
AP136	35 DAP	53.0a	4.3a	22.0ab	0.9a
AP136 + Pectin		53.6a	4.9a	25.1a	0.9a
AP193		32.7b	2.5b	13.4c	0.5b
AP193 + Pectin		38.4b	3.6ab	18.1bc	0.6ab
Control		70.6c	13.8b	12.1b	1.7a
Pectin liquid		74.5bc	17.0a	13.5b	1.9a
AP136	55 DAP	92.7a	19.8a	19.4a	2.1a
AP136 + Pectin		95.6a	20.6a	24.7a	2.3a
AP193		57.1c	14.4b	11.5b	1.8a
AP193 + Pectin		78.7ab	16.9a	19.8a	1.8a

**Table 4:** Effect of pectin amendment as a 1% (*w/v*) liquid suspension on soybean yield by *B. velezensis* PGPR strains AP136, AP193 and commercial *B. japonicum* inoculant in the field trial. The mean value in the column followed by the same letter are not significantly different at *p* ≤ 0.05 using Duncan’s multiple range tests. * Plot weights indicated the total pounds harvested from the two center rows of the four-row plot. ** Test weights indicated the number of pounds in one bushel of soybeans.

Treatment	Plot Weight (kg) *	Test Weight (kg) **
Control	4.0ab	16.6ab
Pectin liquid (PL)	4.5ab	16.6ab
AP136 + Pectin liquid (PL)	5.6a	20.4ab
AP136	5.6a	20.8a
AP193 + Pectin liquid (PL)	3.9ab	16.5ab
AP193	3.5b	12.5b

**Table 5:** Effect of orange peel (OP) amendment on soybean growth promotion and nodulation by *B. velezensis* (Bv) PGPR strains with *B. japonicum* inoculant in the greenhouse trial (OP—orange peel). The mean value in the column followed by the same letter are not significantly different at *p* ≤ 0.05 using Tukey’s multiple comparison tests (mg—milligram).

Treatment	Shoot Length (cm)	Root Length (cm)	Mean Nodule Numbers	Dry Nodule Weight (g)
Control	46.8b	22.4b	11.6d	0.02b
OP 1 mg	55.1ab	25.8ab	17.2cd	0.03b
OP 10 mg	64.6a	26.0ab	15.1cd	0.03b
AP193	58.1a	27.7ab	27.2abc	0.05ab
AP203	65.6a	27.1ab	20.9bcd	0.04ab
AP193 + OP 1 mg	53.7ab	33.7a	39.7a	0.06a
AP193 + OP 10 mg	59.9ab	29.9ab	35.0ab	0.06a
AP203 + OP 1 mg	56.0ab	34.7a	36.5a	0.05ab
AP203 + OP 10 mg	53.6ab	32.6ab	37.7a	0.06a
